# Personalized Hybrid Education Framework Based on Neuroevolution Methodologies

**DOI:** 10.1155/2022/6925668

**Published:** 2022-05-19

**Authors:** Wenjing Yin

**Affiliations:** Zhengzhou Preschool Education College, Zhengzhou, 450000, China

## Abstract

The future pedagogical systems need anthropocentric inclusive educational programs in which the goal should be adjustable according to the knowledge requirements, intelligence, and learning objective of each student. Prioritizing these needs, innovative AI methods are required to assist and ensure the making of conscious educational decisions, in terms of clear identification and categorization with high accuracy of various forms of skills and knowledge of each student. This paper proposes a neuroevolution emerging technique that combines the searchability of evolutionary computation and the learning capability of a hybrid artificial neural networks method. Specifically, the proposed growing semiorganizing neural gas (GsONG) is a practical AI methodology utilizing advanced clustering techniques to enhance the learning experience by categorizing the true abilities, skills, and needs of learners, in an inclusive differentiated learning framework. It is a neural network architecture that includes competing and cooperating neurons with an unstructured mode whereby a cooperation-competition process delimits the topological neighborhood of neurons in a grid to identify patterns for which their classes are not known. To optimize the above process, a heuristic method was used that investigates the space of an objective function by regulating the optimal topologies of neurons that form pathway segments in a semi-contemplative manner. Based on the extensive experiments and results obtained from the GsONG clustering approach, the proposed algorithm can compensate with high accuracy for difficulties in multicriteria grouping and differentiation of uncertainty structures such as in small or tiny data sets.

## 1. Introduction

Inclusive education as a part of differentiated teaching is an organized strategy that is a product of interdisciplinary collaboration and that as a pedagogical approach places at the center the uniqueness of each student, their unique skills, and their needs [1, 2]. It is concerned with the fact that teaching and learning should start from the level of students, instead of being based on a specific and predetermined action plan, which does not include students' readiness, interest, and learning profile [3].

It is essentially the organizational and pedagogical adaptation of inclusive education to meet the various needs of students in mixed grades (lean, mediocre, more capable, and charismatic students) while taking into account the differences of students in terms of readiness, learning style, and interests. In a learning environment characterized by student diversity, each student's personal abilities and strengths are recognized, and he is given opportunities to utilize and demonstrate his various skills through a variety of assessment techniques. It is important to say that differentiated teaching in a learning environment where there is a diversity of students is properly organized and planned and in no way is a product of a random or unbalanced process of allocating learning resources [4].

In this sense, inclusive differentiated teaching is more qualitative than quantitative, with the emphasis on the fact that some students are not given more work than others but that the work is tailored to their specific needs. In this context, interconnected and adequately planned educational activities are offered based on the uniqueness of each student, their skills, and their level. In this way, it is possible to have multiple approaches to the content, the course, and the result of the educational process.

As appealing as teaching differentiation is, it is true that in order for this differentiation to occur and work in a classroom, the curriculum must be adapted. Achieving it is directly related to the functional ways in which students' critical thinking development can take place, as well as through the opportunities offered to students to demonstrate what they have learned [5]. In conclusion, effective differentiated teaching utilizes various methods, means, and materials manage to meet the special needs of all students by increasing their learning opportunities, offering a comfortable and positive environment, where all students achieve high academic achievements [6].

The aim of this paper is to propose a technological system that utilizes computer intelligence algorithms to facilitate decision-making related to curriculum adaptation and categorization of students, based on individual assessments of their unique characteristics. This methodology provides the technological background for automated differentiation of teaching, taking into account characteristics such as the treatment of each student as a person with a history of learning in and out of school, modeling of student metacognitive development, linking to prior knowledge, and building knowledge with gradual progress, according to the perceiving level of each student [7]. Specifically, a differentiated learning framework is proposed, which, with the extensive use of machine learning algorithms, as well as optimization methods, creates a clear framework for multicriteria assessments, to classify students into small groups. The students belonging to each group will have uniform features of learning ability, difficulties in the cognitive object, and their psychosocial and perceptive summary.

The objective of the learning activity determines whether to form groups with students of comparable or mixed ability. If the goal of a group learning activity is to assist struggling pupils, research reveals that heterogeneous groups are the most effective. On the other side, homogeneous grouping might be preferable if the goal is to promote medium-ability groups to learn at high levels. In an educational setting, homogeneous grouping is defined as grouping students of similar instructional levels together so that they can work on materials that are best suited to their individual strengths and areas for improvement. Assessment and instructor observation are frequently used to identify these ability levels [8]. Homogeneous groups enable teachers to create lesson plans that are suited to their students' skills and save time by addressing individual requirements. In any event, because student ability levels differ by subject, categorizing students based on their skills ensures that they are ranked appropriately even within homogeneous groups [7].

On the other hand, heterogeneous groups are preferable if the goal is to raise difficult pupils and help them develop the independence and collaborative skills that come with reduced reliance on the teacher. However, this technique will reduce the value of the exercise for talented and at-grade pupils, even if they will have the opportunity to practice communication and leadership abilities. While some bright children enjoy the benefits and responsibilities that varied groups offer, others dislike them. Knowing the hierarchy of competency and talents, as well as what their peers are capable of, such students are aware that they may be responsible for a bigger portion of the work while dealing with apathetic or disruptive group members. Above-grade-level pupils are unlikely to be challenged by an exercise and will instead become annoyed by everyone's inability to accomplish it as well or as quickly as they could. While they will have the opportunity to develop other talents, they will miss out on the opportunity to learn as much as possible from the task at hand. In the worst-case situation, if a student is very spirited or requires regular stimulation, they may become disruptive and obstruct the learning of the rest of the group or perhaps the entire class. There is a danger that pupils in grade level and below will take a back seat in heterogeneous groups and allow their more gifted classmates to do all the work. This is especially true if the more capable students have assertive personalities, a strong desire to complete the assignment as quickly as possible, and a dismissive attitude toward their peers' talents and efforts.

The proposed system can be used to implement individualized teaching programs in homogeneous learning groups, based on the diversity that characterizes each student and person in general.

## 2. Related Literature

The related literature of the proposals on the educational methods to provide differentiated and personalized training mainly focuses on theoretical proposals. The potential of being able to provide personalized training in the educational process is great because it gives to the learner the best possible learning experience. Personalized e-learning encompasses a variety of educational technology and pedagogical methods that take into account individual student variations [4] and can customize the generic virtual training environment to meet their own needs. The competence of professors and educational material quality are key factors in e-learning programs [9], but instructors' competency is revealed through their approach, teaching style, and assistance throughout the entire online education experience. All these elements contribute to the positive influence on students' emotions and subsequently trigger flow. Ardura and Artola [10] intend to contribute to the flow and fill the hole that has been identified in terms of knowledge possessed in personalized e-learning settings in their extensive investigation. They also look at how education-related and subjective factors interact to create flow in a personalized e-learning environment, as well as evidence supporting the moderating influence of individual differences in gender and academic achievement. Even though this is detailed research, the limitations are that the timeframe for the calculations is limited making it impossible to say if the reported associations will alter over time as new e-learning methods for customization emerge. Furthermore, the sample of the study is limited to just one online college leaving the field open for contribution and enhancement of this model and the generalization of the results through further study.

In his paper, Nganji [11] provides a concept for a learning environment that allows learners to create their own online learning spaces and engage with pooled materials. This model is called Flexible and Accessible User Constructed Learning Environment (FAUCLE) and essentially is a learner-centered model that connects the elements that contribute to the e-learning process and creates relationships among them. These are the learner, the educator, the accessibility expert, the e-learning programmer, the learning environment, and the learning content apps. The prerequisite for this model to take place is for the institution that provides the training to make applications and other resources available to these students in a form that is both accessible and useable, allowing them to create their learning environments with flexibility. The fact that this model is theoretical means that it needs to be implemented and tested to yield empirical data that can be compared to other studies. Milicevich and Ivanovic [7] in their work aim to address the research by examining aspects of tailored e-learning, as well as intelligent and interactive technologies. They show online education systems that are currently state-of-the-art that are powered by artificial intelligence at the end. Their study is also theoretical and serves as a suggestion for institutions and organizations who want to adopt these new technologies and approaches in e-learning. Maghsudi et al. [12] in their study give a quick rundown of current research, look into the challenges of AI/ML-based individualized education, and propose possible solutions. They conclude that “personalized education” is one of artificial intelligence's most valuable educational merits because it significantly improves education quality in several dimensions by adapting to the unique characteristics and expectations of each learner, such as personality, talent, objectives, and background. In addition, in unusual circumstances such as the COVID-19 outbreak [13] or natural disasters, online teaching is invaluable. Indeed, traditional education requires substantially more resources than online education in terms of classroom space, scheduling, and human resources, making it vulnerable to failure in the event of even minor changes in circumstances. As a result, new alternatives are unavoidable [14, 15]. Personalized education, despite the potential for a dramatic shift from traditional to modern education paradigms, is fraught with difficulties.

From the above literature, the conclusion is that the research community focuses on finding ways to combine technology with the traditional educational processes to provide a differentiated experience. What this paper does is go a step further and propose a practical methodology utilizing machine learning to enhance the learning experience.

## 3. The Gsong

To create an intelligent framework for categorizing the true abilities, skills, and needs of learners, a differentiated learning framework is proposed, which makes extensive use of nonsupervised machine learning technologies to achieve its goals [16]. Unsupervised learning is the ability to identify patterns for which their classes are not known, and the system generates predictions based on some distribution or some quantitative measures to evaluate and characterize the similarity of data in corresponding groups called clusters. The general clustering technique [17] is based on the idea that a data set *D*={*x*_1_,  *x*_2_,…, *x*_*n*_}*F*, where *x*_*i*_=(*x*_*i*1_,  *x*_*i*2_,…, *x*_*ir*_) is a characteristic of the set *X* ∈ *R*^*r*^ and *r* is the number of dimensions in data. After defining the set of blocks K, assign each point *x*^(*i*)^ of the data set to a block *C*_*k*_ so that the *S*_*core*_(*C*,  *D*) is maximized or minimized as appropriate. How to calculate a function of this type is given by the following formula [17]:

(1) $$\begin{array}{c} S_{\text{core}}\left(C,\;D\right)=\sum_{k=1}^{K}d\left(x,c_{k}\right), \end{array}$$

where *c*_*k*_=1/*n*_*k*_∑_*x*∈*c*_*k*__*x* and *d*(*x*, *y*)=‖*x* − *y*‖^2^.

Elements that belong to a cluster show greater or relatively greater similarity. The training of a computational intelligence model with the method of clustering is called to calculate and finally classify into clusters, data *x*_1_,…, *x*_*n*_ without giving the values *f*(*x*_1_),…, *f*(*x*_*n*_). In this work, the proposed model applies clustering based on competitive learning and is specifically implemented using artificial neural networks [14, 18].

Specifically, the methodology provides for the classification of students into homogeneous groups based on their particular characteristics [19]. For this reason, a neural network was created that includes a competitive layer of competing neurons. Each competing neuron *i* is characterized by a weight vector *w*_*i*_=(*w*_*i*  *d*_,…,*w*_*i*  *d*_)^*T*^, *i*=1,…, *M*, and computes a similarity measure among the input information *x*_*i*_=(*x*_*i*  *d*_,…,*x*_*i*  *d*_)^*T*^*x* ∈ *R* and in *w*_*i*_ weights vector. Each time a student's characteristics appear as an entry in the network, competition is created between competing-level neurons to identify the winning neuron whose weight vector shows the greatest similarity to that input. The winning neuron *m* sets the output of *o*_*m*_=1, while the other neurons give *o*_*i*_=0, where *i*=1,…, *M* and *i* ≠ *m*. As a measure of similarity to find the winner neuron, a function inversely proportional to the Euclidean distance ‖*x* − *w*_*i*_‖ of the input vector *x*^*n*^ from the vector of *w*_*i*_ weights was used. Therefore the proposed neural network implements a representation of the input *x*, dimension *d*, in the coordinates of the grid *r*_*m*_=(*z*_*m*1_,  *z*_*m*2_)^*T*^.

More specifically, the proposed neural system forms a self-organizing map of its structures, starting from the process of initializing the weights *w*_*i*_=(*w*_*il*_,…,*w*_*i*  *d*_)^*T*^. To achieve this, small weight values generated by a random number generator are given. The weight table of the hidden layer H is calculated as follows [20]:

(2) $$\begin{array}{c} Η=g\left(\omega x+b\right). \end{array}$$

The output weights *β* are calculated based on the following function:

(3) $$\begin{array}{c} \beta ={\left(\frac{Ι}{C}+H^{T}H\right)}^{-1}H^{T}X, \end{array}$$

where *H*=[*h*_1_,…, *h*_*N*_] is the hidden level outputs and *X*=[*x*_1_,…, *x*_*N*_] is the input data. *β* can also be calculated from the general relation as follows:

(4) $$\begin{array}{c} \beta ={Η}^{+}T. \end{array}$$

After this initialization, there are three basic procedures:

*Competition*. For each *x*^*n*^, the grid neurons calculate the similarity function. The winner is the neuron with the highest similarity value. The Euclidean distance between *x*=(*x*_1_,…, *x*_*d*_)^*T*^*x* ∈ *R* and *w*_*i*_=(*w*_*il*_,…,*w*_*i*  *d*_)^*T*^ of the opposing neurons is used as a function of similarity.

*Cooperation*. The winner-neuron delimits the topological neighborhood of neurons in the grid, which will adjust their weights to the input vector. *h*_*j*,*i*_ denotes the topological neighborhood centered on the winner-neuron *i*, which includes a set of neurons, one of which is denoted as *j*. Also denoted by *d*_*j*,*i*_ is the distance between the winner neuron *I* and a neuron *j*. Thus, the topological neighborhood satisfies the above constraints [17, 21].

(5) $$\begin{array}{c} h_{j,i}\left(x\right)=\exp\left(-\frac{d_{j,i}^{2}}{2\sigma^{2}}\right), \end{array}$$

where parameter *σ* is the topological neighborhood's effective width, which defines how many neurons in the winner's neighborhood participate in the training process. This parameter decreases in each season *n* at an exponential rate according to the relation [21, 22]:

(6) $$\begin{array}{c} \sigma \left(n\right)=\sigma_{0}\exp\left(-\frac{n}{\tau_{1}}\right),\;n=0,1,2,\ldots , \end{array}$$

where parameter *σ*_0_ is the initial value of the active amplitude and *τ*_1_ is the polarity constant of the network.

*Synaptic Adaption*. In this last stage of the training process, the weights of the competing-level neurons are updated. The amount of this change is given by the following relation [23, 24]:

(7) $$\begin{array}{c} \Delta w_{j}=\eta h_{j,i}\left(x\right)\left(x-w_{j}\right), \end{array}$$

where *i* is the winning neuron and *j* is the neuron in the neighborhood of *i*. Finally, given the vector of weights *w*_*j*_(*n*) for a given time *n*, the new vector for the time *n*+1 can be calculated from the following relation:

(8) $$\begin{array}{c} w_{j}\left(n+1\right)=w_{j}\left(n\right)+\eta \left(n\right)h_{j,i}\left(x\right)\left(n\right)\left(x\left(n\right)-w_{j}\left(n\right)\right). \end{array}$$

From the above relation, it follows that the learning rate *η*(*n*) depends on time. More specifically, it starts from an initial value of *η*_0_ and decreases exponentially with increasing time *n* [25]:

(9) $$\begin{array}{c} \eta \left(n\right)=\eta_{0}\exp\left(-\frac{n}{\tau_{2}}\right),\;n=0,1,2,\ldots , \end{array}$$

where *τ*_2_ is the polarization constant of the network.

In addition, the above process is divided into two phases:

*Ordering phase* is the initial phase, and it is during this phase that the competing-level weights are topologically arranged. During this phase, the learning rate *η (n)* starts from a value around 0.1 and gradually decreases, up to the value of 0.01. These values are achieved as follows:

(10) $$\begin{array}{c} \eta \left(n\right)=\eta_{0}\exp\left(-\frac{n}{\tau_{1}}\right),\;n=0,1,2,\ldots ,\text{ with }\eta_{0}=0.1\text{ and }\tau_{2}=1000. \end{array}$$

The following network polarization values were used to calculate the learning rate and active amplitude values in each iteration:

(11) $$\begin{array}{c} \tau_{2}=\frac{n_{0}}{lnln\left(100\cdot \eta_{0}\right)\;}\text{ and }\tau_{1}=\frac{n_{0}}{lnln\;\left(\sigma_{0}\right)\;}, \end{array}$$

where *n*_0_ is the number of repetitions of the phase of the device, *η*_0_ is the initial learning rate, and *σ*_0_ is the initial value of the active amplitude that in turn results from the following relation:

(12) $$\begin{array}{c} \sigma_{0}=\sqrt{w^{2}+h^{2}}, \end{array}$$

where *w* and *h* are the length and height of the two-dimensional grid, respectively.

Also, the topological neighborhood function *h*_*j*,*i*(*n*)_ initially includes almost all competing-level neurons centered on the winning neuron and is gradually limited to a few neurons or even just the winning neuron. Considering a two-dimensional frame, the value of the “radius” of the grid was taken as the initial value *σ*_0_ of the active width and as the value of the parameter *τ*_1_ of the above relation [26]:

(13) $$\begin{array}{c} \sigma \left(n\right)=\sigma_{0}\exp\left(-\;\frac{n}{\tau_{1}}\right),\;n=0,1,2,\ldots \;with\;\tau_{1}=\frac{1000}{\log\log\sigma_{0}}. \end{array}$$

*Convergence phase* is the phase in which the weights acquire their final values better coordinated in the training examples. In this phase, the number of repetitions was determined by the dimension of the network inputs. The learning rate *η*(*n*) remained constant at values close to 0.01, and finally, the neighborhood *h*_*j*,*i*(*n*)_ was limited to the nearest neighbors of the winner-neuron, ending up containing only the winner neuron.

In the second phase (convergence), the values of the learning rate and the active amplitude remained constant and equal to 0.01 and 0.0001, respectively.

Regarding the learning rate, the variables that were selected are *λ*_*i*_ and *λ*_*f*_ that control the rate at which the neural network learns, while *ε*_*i*_ and *ε*_*f*_ define the initial and final rate according to which the neural network is trained. The variable *t*_max_ is the maximum number of execution times. With *t* the current season, *t*_max_ the total number of seasons, $\overset{⟶}{x}$ the input signal generated at the beginning of each season, *n* each network node, *n*_*w*_ the vector that carries each neuron, and *k* is the degree of each node once, it has been classified into steps. All nodes were sorted in ascending order based on the Euclidean distance of their vectors from the input signal as follows [21, 26]:

(14) $$\begin{array}{c} {\left\|\overset{⟶}{x}-\overset{⟶}{n_{w}}\right\|}^{2}. \end{array}$$

The weights of the nodes were adjusted in the order we have arranged them so that [27]

(15) $$\begin{array}{c} \overset{⟶}{n_{w}}\;\leftarrow \;\overset{⟶}{n_{w}}+\left[\overset{⟶}{n_{w}}\;\times e\left(t\right)\times h\left(k\right)\times \left(\overset{⟶}{x}-\;\overset{⟶}{n_{w}}\right)\right], \end{array}$$

where

(16) $$\begin{array}{c} h\left(t\right)=\exp\;\left(\frac{-k}{\sigma^{2}\left(t\right)}\right),\\ \sigma^{2}\left(t\right)=\lambda_{\iota }\times \;{\left(\frac{\lambda_{f}}{\lambda_{i}}\right)}^{t/t_{\max}},\\ \varepsilon \left(t\right)=\varepsilon_{\iota }\times \;{\left(\frac{\varepsilon_{f}}{\varepsilon_{i}}\right)}^{t/t_{\max}}. \end{array}$$

To optimize the above process, a heuristic method was used that investigates the space of an objective function by regulating the optimal topologies of neurons that form pathway segments in a semi-contemplative manner. Each neuron is attracted to the position of the best location found by the heuristic function and the best location it has encountered, while, at the same time, it tends to move randomly.

Specifically, when an entity *i* discovers a locality that is superior to the previous ones it located, then it upgrades it to the best current for *i*. There is a current best for all *n* entities at any time *t*, during iterations. The goal is to find the best overall until the position of the neuron can no longer be improved [28].

Let *p* and *u* be the position and velocity for entity *i*, respectively. The new velocity vector is identified by the following formula [29]:

(17) $$\begin{array}{c} u_{n,m}^{\text{new}}=u_{n,m}^{\text{old}}+\Gamma_{1}\times r_{1}\times \left(p_{n,m}^{{\text{local}}_{\text{best}}}-p_{n,m}^{\text{old}}\right)+\Gamma_{2}\times r_{2}\times \left(p_{n,m}^{{\text{global}}_{\text{best}}}-p_{n,m}^{\text{old}}\right), \end{array}$$

where *u*_*n*,*m*_ represents the convergence speed, *r*_1_, *r*_2_ represents independent random numbers, Γ_1_, Γ_2_ represents learning parameters, *p*_*n*,*m*_^local_best^ represents the best local solution, and *p*_*n*,*m*_^global_best^ represents the best total solution.

**Figure 1:**
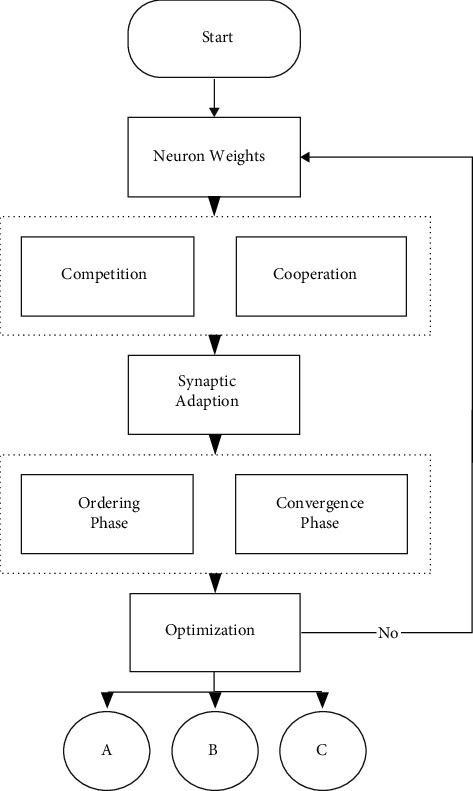
The logical data flow of GsONG.

The heuristic optimization algorithm renews the convergence speed component and then adds speed to the position component. This renewal depends on both the optimal solution/position discovered and the one used by all active neurons. If, at some point, the best solution discovered is better than that of the population, it replaces it. The initial locations of all neurons consist of being evenly distributed so that they are a sample for most areas of the search space. It is also possible for the original vector of an entity to be taken as zero. The new location is described by the following equation [22, 29] (Figure 1):

(18) $$\begin{array}{c} p_{n,m}^{\text{new}}=p_{n,m}^{\text{old}}+u_{n,m}^{\text{new}}, \end{array}$$

where *u* is delimited to a range [0, *u*_max_].

A descriptive illustration of the overall process is presented in the diagram below.

## 4. Experiments

The aforementioned algorithm was applied to student assessment data to handle the problem of categorizing students into heterogeneous groups with comparable features at the group level, where we assume that we have students who should be classified into groups at most.

The data used relate to quantitative individual performance and psychosocial data of students of a heterogeneous class of students, to implement the multicriteria test procedures.

Specifically, the data refer to the holistic assessment of an elementary school student class with a total capacity of 21 people, where Raven's IQ test was used to assess general mental ability (V1), and the math performance test for primary school students was used to determine mathematical skills (V2). The student's grade point average was used to evaluate performance (V3). The learning disability scale was used to determine social or emotional skills or difficulties (V4). Finally, the psychosocial adjustment tool was used to assess social or emotional skills or difficulties (V5). It is important to emphasize that the problem is trying to be identified on a completely realistic basis, based on the real process that a teacher would follow in a department applying differentiated learning but within the children who are already part of a department. Specifically, the 21 children who participate in this classification process based on the proposed system are a real school class.

Specifically, the 21 children who participate in this classification process based on the proposed system are a real school class. The separation attempted is a realistic approach where the groups of 3, 4, 5, or 6 children that may arise are a fully satisfactory sample of children with homogeneous elements to whom personalized learning techniques can be applied. It should be emphasized that the resulting groups do not form new classes, but groups that receive training materials, instructions, exercises, and so on depending on the level in which they were classified but all within the same classroom.

In order to prove the correct use of this data set, a thorough preprocessing of the data was performed for the purpose of validation checks that prove the reliability of the data set under consideration, before the use of the proposed algorithm. This process is necessary as the initial data often suffer from various kinds of problems, such as conflicting information, coding inconsistencies, noise, and extremes, but also in addressing specific requirements that require data transformation, such as the discretization, the normalization, the reduction of their dimensions, or the selection of the most appropriate characteristics.

Initially, an indicative statistical analysis of the data set was performed. The main object of the above statistical analysis is the analysis and interpretation of the data used with the ultimate goal of drawing safe conclusions for making correct decisions. Specifically, Table 1 shows the probability for each sample to belong to a specific subset and if the sample space is made up of discrete random variables for which a cumulative probability function can be used to determine the distribution. The statistical analysis of all 21 students is presented in Table 1.

**Figure 2:**
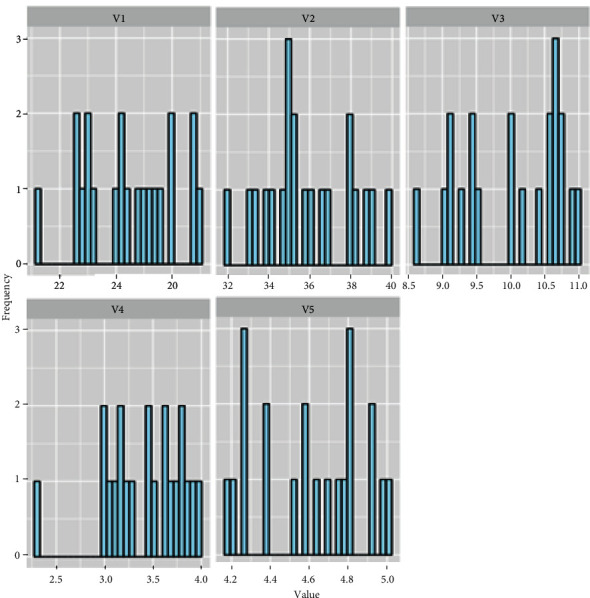
Histogram for each feature of data set.

For the clear and distinct localization of the fluctuation of their values, the graphs of the statistical frequencies of the price ranges of each feature used in the data set are presented in histograms. The height of each region is equal to the ratio of the frequency to the range of values represented by the rectangle. All five features are presented in Figure 2.

**Figure 3:**
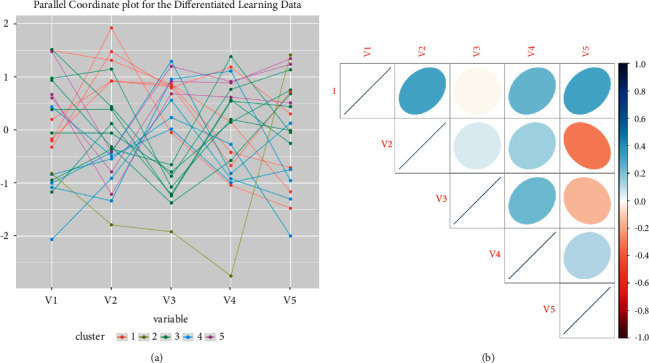
(a) Parallel coordinates plot between features and (b) correlation matrix between features.

Because the data set used is multivariate numeric data, the parallel coordinates plot is listed in Figure 3(a), which is an imaging technique that facilitates the comparison of multiple quantitative variables simultaneously in order to identify patterns, similarities, complexes, and positive and negatives or neutral data relationships.

To investigate the two-way relationships between the features of the data set, a correlation analysis was performed, and Figure 3(b) shows the resulting correlation matrix.

**Figure 4:**
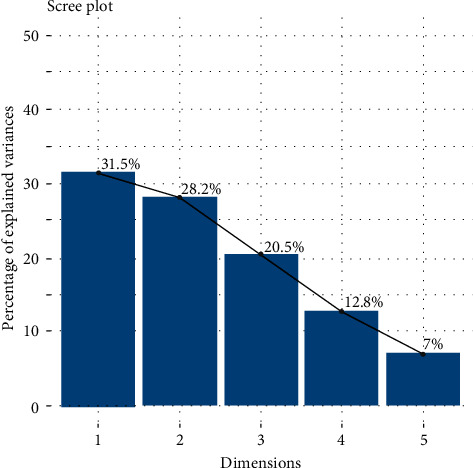
Principle component analysis.

Correlations are useful because they can indicate a predictive relationship that can be exploited in practice, although statistical dependence is not sufficient to prove the presence of a causal relationship (i.e., the correlation does not imply causality). A principle component analysis (PCA) test was then performed to detect data covariance and to apply if parameter reduction is required. As can be seen from the scree plot in Figure 4, the principal components retain less than 60% of the statistical data from the original data, so no parameter reduction is required.

**Figure 5:**
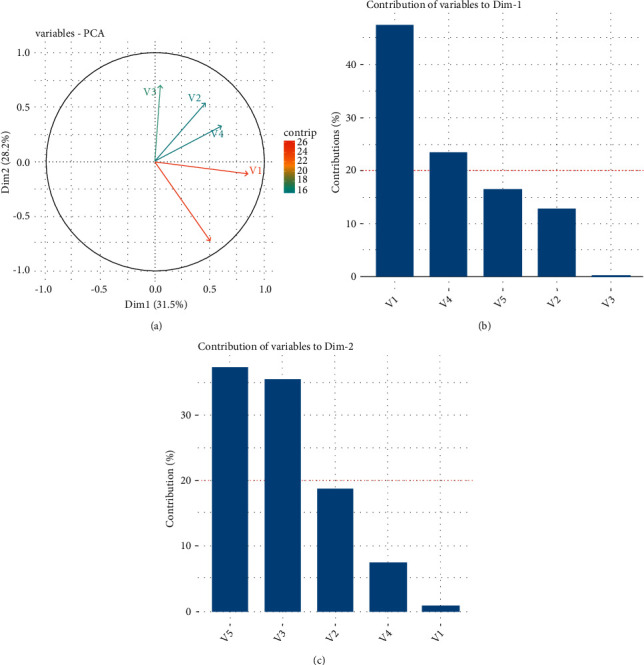
(a) Plot of contribution of PCA, (b) plot of contribution Dim-1, and (c) plot of contribution Dim-2.

From Figures 5(a)–5(c), it is understood that V1 and V2 tend to increase together in the first dimension, while in the second dimension, V1, V2, and V3 increase together. These two groups of features have a homogeneous and corresponding correlation.

**Figure 6:**
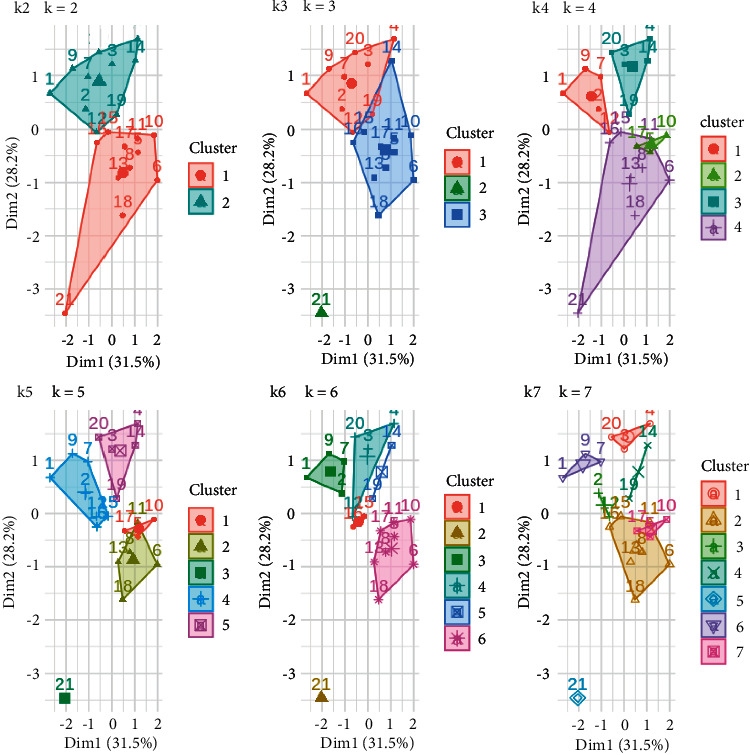
Plot of *k* = [2–7] centers.

The process of pretreatment of the set performed proves and ensures the quality of the data to be used by the proposed algorithm. Then, in order to identify the appropriate groups of students that will be the uniform clusters of differentiated learning, sequential analyzes were performed with various clustering methods (such as k-means, k-Medoids, and k-Centroids) [17, 22]. Specifically, successive configurations were performed with the available data, from 2 to 7 groups (clusters centers), in order to identify the best. For example, in the example of Figure 6 where he presents the configurations using the k-means algorithm, as this algorithm is sensitive to the initial positions of the centers of the clusters, 10 initial configurations were created, and then, all the results were calculated on average.

**Figure 7:**
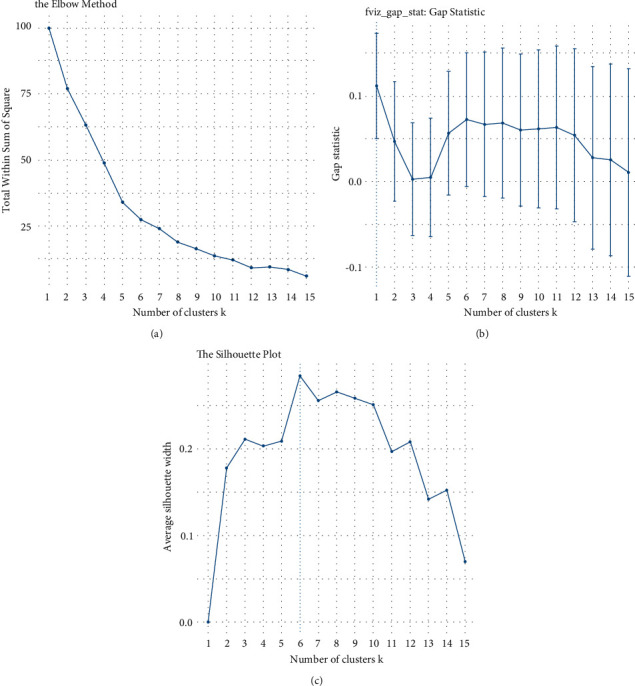
(a) “Elbow” method, (b) “Gap” statistic, and (c) “Silhouette” method.

The above visual assessment gives clear explanations of where the demarcations between clusters occur; however, no information is given on the optimal number of clusters. To determine the optimal number of clusters, the method “Elbow” was used sequentially, in which the sum of the squares for each number of blocks is calculated and formed and the optimal number results in the abrupt change of inclination (Elbow), as in Figure 7(a).

“Gap” statistics method is also used that compares the total variance within clusters for different center values with their expected values under zero data reference distribution. The estimation of the optimal clusters is the value that maximizes the statistical element of the gap, that is, that gives the largest statistical gap, which means that the clustering structure is far from the random uniform distribution of points. The “Gap” statistic is shown in Figure 7(b).

The “Silhouette” method was also used that calculates the average silhouette of the observations for different clusters values. The optimal number of blocks is the one that maximizes the average silhouette in a range of possible values. The “Silhouette” method is shown in Figure 6(c).

**Figure 8:**
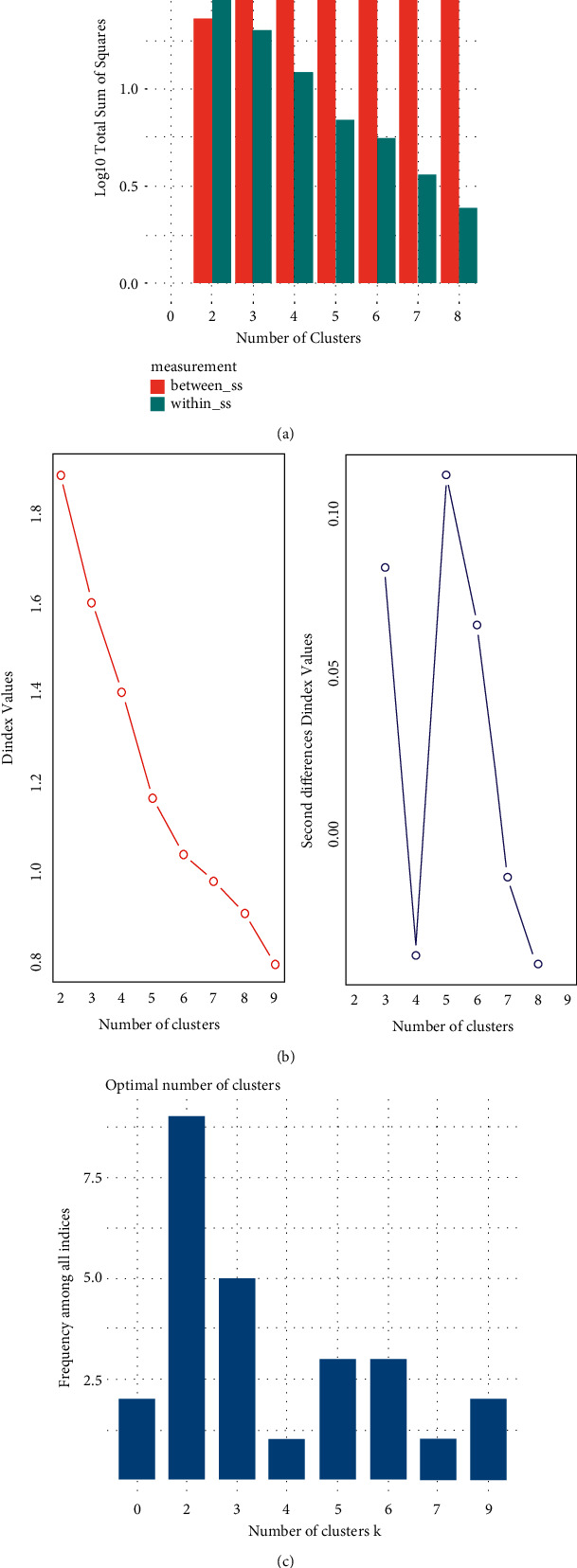
(a) Sum of squares method, (b) Clustree method, and (c) NbClust method.

Another validation method used is to select the optimal number of clusters by minimizing the sum of squares within a cluster (how tight each cluster is) and by maximizing the sum of squares between the clusters (how sparsely the clusters are distributed). This method is shown in Figure 8(a).

Also, the Clustree statistical method produces a single score that takes into account only one set of clusters at a time considering how the samples change groups as the number of clusters increases. This is useful for showing which groups are different and which are unstable. The methodology is shown in Figure 8(b).

Another very interesting measurement comes from the NbClust method for determining the relative number of clusters, which proposes the best scheme from the different results obtained from the evaluation of 30 indicators.

The specific measurement is shown in Figure 8(c).

A Davies–Bouldin index was used to evaluate the candidate solutions [17, 22]. We consider that *R*_*i*,*j*_ is an evaluation measure of each cluster that is calculated by the following equation:

(19) $$\begin{array}{c} R_{i,j}=\frac{s_{i}+s_{j}}{M_{i,j}}, \end{array}$$

where *s*_*i*_, *s*_*j*_ are the dispersions of the *i* and *j* blocks, respectively, which are calculated from the following equation:

(20) $$\begin{array}{c} s_{i}={\left(\frac{1}{T_{i}}\sum_{j=1}^{{Τ}_{i}}{\left|X_{j}-A_{i}\right|}^{q}\right)}^{1/q}, \end{array}$$

where Τ_*i*_ is the number of vectors in the *i* block, *X*_*j*_ is the vector of each student's attributes, and *A*_*i*_ is the center of the *i* block. Each student is classified in the cluster whose center is closer to his own. *M*_*i*,*j*_ is the Minkowski metric for the distance of *i* and *j* blocks, which are calculated from the following equation [21, 30, 31]:

(21) $$\begin{array}{c} M_{i,j}={\left\{\sum_{k=1}^{N}{\left|a_{k,i}-a_{k,j}\right|}^{p}\right\}}^{1/p}, \end{array}$$

where *a*_*k*,*i*_ is the *k* element of *A*_*i*_ and *a*_*k*,*j*_ is the *k* element of *A*_*j*_. The value of the objective function is finally defined as follows:

(22) $$\begin{array}{c} \underline{R}=\frac{1}{N}\sum_{i=1}^{N}R_{i}, \end{array}$$

where *R*_*i*_ is the maximum value of *R*_*i*,*j*_ for *i* ≠ *j*.

If a group of students was too small or too large, an error was introduced into the objective function, doubling its value, making all groups four to six students.

The number of participants in a collaborative student group should be between four and six, as this allows good cooperation and communication among the members.

As a result, *k* was calculated as an integer consistently greater than or equal to the quotient of dividing the number of students by the number four. Following the completion of the group separation using the suggested algorithm, the solutions were assessed intragroup for homogeneity using the coefficient of variation (CV).

The CV is an index of relative variance or dispersion, which expresses the homogeneity of a set of measurements of values of a random quantitative variable and the accuracy of an experimental design.

The following ratio was used to calculate the index for sample data:

(23) $$\begin{array}{c} CV=\frac{S}{\underline{Y}}100, \end{array}$$

where *S* is the standard deviation and *Y* is the arithmetic mean of the sample measurements.

Values close to zero indicate homogeneity in terms of characteristics, while values close to 1 indicate inhomogeneity. In general, the values of the CV index show the level of homogeneity as follows:

1. High (0.00 < CV ≤ 0.25)
2. Medium (0.25 < CV ≤ 0.40)
3. Low (CV > 0.40)

The Kruskal–Wallis test [32] was used to determine if the resulting groups differed at different levels of statistical significance (0.01, 0.001).

The nonparametric Kruskal–Wallis test was used for the nonparametric analysis of variance in independent samples and was selected as in this case, the condition of normality of the populations examined is not met, the sample is small (<20 for each cluster), and the values of the dependent variables do not express quantity but are ranks where individuals are simply ordered according to some criterion.

The way to calculate the acceptance or rejection of the null hypothesis that the random samples are homogeneous is to examine whether the quantities *R*_*i*_/*n*_*i*_, *i*=1,…, *k*, *k* > 3 are approximately equal to each other and equal to (*n*+1)/2 or if the following ratio is close to zero:

(24) $$\begin{array}{c} \sum_{i=1}^{k}{\left(\frac{R_{i}}{n_{i}}-\frac{\left(n+1\right)}{2}\right)}^{2}. \end{array}$$

The magnitude of the size effects was calculated using the Eta Squared *η*^2^ and Cohen's *d* indicators [33]. Implementing the clustering process, initially, the parameters of the proposed algorithm were randomly initialized, based on the description performed above.

To find the optimal values for which the algorithm performs best, extensive trial and error tests were performed for different hyperparameters of the optimization algorithm. Initially, the population of optimal solutions was tested by testing values from 10 to 150 with a progressive increase of 10 units.

**Figure 9:**
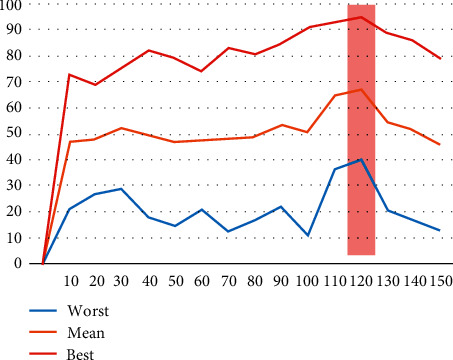
Optimization parameters.

The algorithm performed 10 iterations for each value, whose diagrammatic representation of the results is presented in Figure 9, where the best, worst, and average values of their results are stated.

As it is understood, the optimal parameters that the algorithm shows greater convergence are for 120 particles, which were selected for the further clustering process. Also, Table 2 presents in detail the above values.

**Figure 10:**
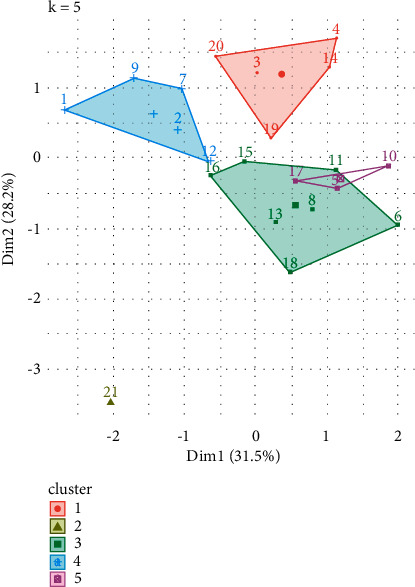
Clusters distribution.

The resulting clusters are shown in Figure 10.

**Figure 11:**
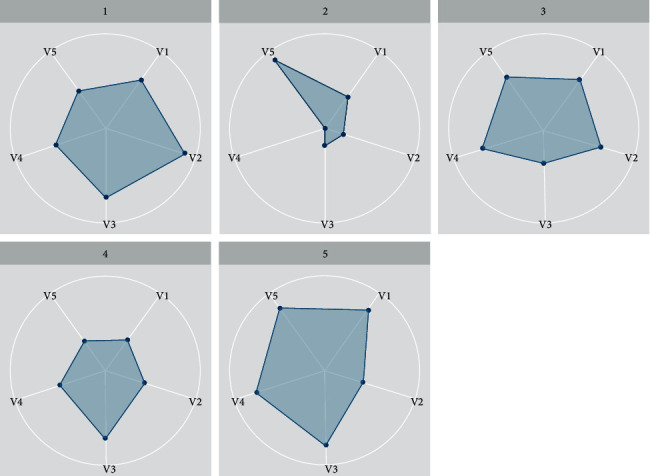
Differentiated learning attributes.

Also, Figure 11 shows the exact distribution of the differentiated learning attributes.

The clusters created by the proposed algorithm with the average values per rating scale are presented in Table 3.

The values of the CVs, for the evaluation of the homogeneity within each cluster formed through the proposed algorithm, yielded values from 0.00 to 0.06, which are presented in Table 4.

Finally, the results of the Kruskal–Wallis test showed that the five groups differ significantly in [21]:

1. The general mental capacity (V1), [*χ*^2^(3)=18,67, *p* < 0,001, *d*=6,9, *η*^2^=0,92] 
2. Mathematical performance detection test (V2), [*χ*^2^(3)=18,23, *p* < 0,001, *d*=5,9, *η*^2^=0,90]
3. The quarter points that are an estimate of their performance (V3), [*χ*^2^(3)=17,62, *p* < 0,01, *d*=4,9, *η*2=0,86]
4. The scale of detection of learning difficulties (V4), [*χ*^2^(3)=19,26, *p* < 0,001, *d*=9,4, *η*^2^=0,96] 
5. Their psychosocial adaptation characteristics (V5), [*χ*^2^(3)=16,92, *p* < 0,01, *d*=4,3, *η*^2^=0,82]

## 5. Discussion and Conclusions

Based on the results obtained and presented in detail above, it is obvious that the utilization of the proposed algorithm can find a reliable solution to the extremely difficult problem of creating and forming student groups for the implementation of individualized teaching programs. The methodology proved that through the widespread use of intelligent methods, small and heterogeneous groups of students can emerge with the members of each group sharing similar features in terms of student ability [34], learning challenges [35], and psychosocial and cognitive profile [36]. In this way, in addition to being able to quickly manage the student potential in their class and knowing the individual characteristics of each group, the teacher can easily manage the student potential of their class [37]; he can offer high-quality education, through differentiated approaches that take into account the special educational needs and capabilities of each group, their particular interests, their unique experiences, their learning rhythms, their learning style, their cultural background, and their self-perception [38]. Also, as a clustering approach, the algorithm can be used in both traditional classrooms and digital or e-learning programs, facilitating the educational role [39], as it can compensate for difficulties in multicriteria grouping and differentiation of students in a variety of subjects [40]. Another significant benefit of the method is that it may be used with a large number of students and deliver results in a short period of time, provided of course there is the appropriate data for processing [41]. Another supporting presumption is that there is no limit to the data that can be accepted as quantitative data or to the evaluable factors that result from the multifaceted and holistic assessment of the student [42].

In this study, one limitation was the small number of participants, which may raise validity issues. However, the algorithmic approach used was weighted to compensate for any psychometric issues, and it is important to emphasize—which gives the method applicability in real conditions—that the application of computational methods was done in real order contexts, where there are physical limitations to a maximum number of students attending them.

From a technical point of view, the algorithm presented, which is proposed for the first time in the literature, shows a very high degree of convergence, which is evidenced by the very high clustering results that were achieved and confirmed experimentally. A very important observation also concerns the fact that the optimization method used converges very quickly, while, in all the tests, it was not observed to be trapped in local optimal, thereby avoiding incorrect cluster formations. The principle of differentiated learning is a modern educational method that aims to offer high-quality education, through differentiated approaches that take into account the special educational needs and capabilities of each student, their special interests, their unique experiences, their learning rhythms, their learning style, their cultural background, and their self-perception. Even, in this case, however, the level of students is never the same, resulting in the adaptation of teaching to the different levels of learning ability that exist within a classroom. The internal differentiation required in these cases should include a wide variety of practices and individualized forms of organizing the learning process. In that vein, this paper presented an innovative and fully efficient differentiated learning framework. It is an intelligent system that can classify students into similar homogeneous groups, based on their general mental ability, the performance of their student skills, grade points, learning difficulties they may face, and finally the criteria of psychosocial adjustment for the assessment of skills and their school adaptation to the school environment. It is based on advanced engineering learning techniques for performing high-level analyzes for the effective reorganization of educational learning systems based on evaluation criteria. The implementation of the proposed algorithm is based on the ideal use and combination, for the first time in the machine learning literature, of the two well-known clustering methodologies (cooperation and competition) in order to produce an extremely efficient and fast neural system. The proposals for the continuation of this research focus mainly on the investigation and extension of the model with inherent capabilities of natural language processing, for the automated system to fully utilize the capabilities of the wider dependencies of modeling learning systems, with greater accuracy and efficiency. The future study of the effect of such a grouping methodology on the student's learning development in comparison to traditional methods of separation is also intriguing, as is the realization of such research using nonparametric machine learning methods.

## Figures and Tables

**Table 1:** Statistical analysis of data set.

	V1	V2	V3	V4	V5
Mean	21.12	32.08	8.63	2.31	4.17
1st Q	23.09	34.72	9.40	2.35	4.38
Median	24.34	35.19	9.21	2.48	4.63
Mean	24.44	35.87	9.04	2.42	4.60
Std	3.45	4.89	0.97	0.49	0.59
3rd Q	25.51	37.82	9.67	2.73	4.81
Max	26	39	10	3	5

**Table 2:** Optimization process results.

Parameters	Worst	Mean	Best
10	21	47	73
20	27	48	69
30	29	53	76
40	18	50	82
50	15	47	79
60	21	48	74
70	13	48	83
80	17	49	81
90	22	54	85
100	11	51	91
110	36	65	93
120	40	68	95
130	21	55	89
140	17	52	86
150	13	46	79

**Table 3:** Mean and standard deviation of clusters.

Cluster		0 (6 studs)	1 (5 studs)	2 (4 studs)	3 (6 studs)	4 (6 studs)
V1	Mean	18.86	15.711	25.23	21.47	21.39
	StDev	0.77	1.18	0.94	1.08	1.07
V2	Mean	30.74	27.69	37.81	32.94	32.96
	StDev	1.02	0.44	0.98	1.35	1.34
V3	Mean	8.42	6.78	9.99	9.43	9.51
	StDev	0.53	0.47	0.03	0.56	0.57
V4	Mean	2.01	1.03	2.98	3.02	3.04
	StDev	0.02	0.46	0.01	0.01	0.01
V5	Mean	3.84	2.79	5.05	4.96	4.89
	StDev	0.77	0.48	0.02	0.04	0.05

**Table 4:** Coefficient of variation of each cluster.

Cluster	V1	V2	V3	V4	V5
0 (6 students)	0.04	0.03	0.06	0.01	0.02
1 (5 students)	0.06	0.02	0.05	0.03	0.01
2 (4 students)	0.04	0.03	0.01	0.01	0.01
3 (6 students)	0.05	0.04	0.05	0.01	0.02
4 (6 students)	0.06	0.05	0.05	0.01	0.02

## Data Availability

The data used in the paper are available upon request.
